# A Transgenic Mouse With a Humanized B-Cell Repertoire Mounts an Antibody Response to Influenza Infection and Vaccination

**DOI:** 10.1093/infdis/jiae472

**Published:** 2024-09-24

**Authors:** Valarmathy Murugaiah, Simon J Watson, Robert F Cunliffe, Nigel J Temperton, Stevo T Reece, Paul Kellam, John S Tregoning

**Affiliations:** Department of Infectious Disease, Imperial College London, London, United Kingdom; Kymab, a Sanofi Company, Babraham Research Campus, Cambridge, United Kingdom; Department of Infectious Disease, Imperial College London, London, United Kingdom; Viral Pseudotype Unit, University of Kent and Greenwich, Chatham, United Kingdom; Kymab, a Sanofi Company, Babraham Research Campus, Cambridge, United Kingdom; Department of Infectious Disease, Imperial College London, London, United Kingdom; Kymab, a Sanofi Company, Babraham Research Campus, Cambridge, United Kingdom; Department of Infectious Disease, Imperial College London, London, United Kingdom

**Keywords:** influenza, antibody, humanized, antigenic sin, universal vaccine

## Abstract

The development of a universal influenza vaccine likely requires an understanding of previous exposure to influenza virus (through vaccination or infection) and how that shapes the antibody repertoire to vaccination, sometimes called original antigenic sin or antigenic imprinting. While animal models can have a much more defined exposure history, they lack a human B-cell repertoire. Transgenic mice with the complete human immunoglobulin locus enable studies of controlled infection history leading to human-like antibody evolution. Here we evaluated responses to influenza in the Intelliselect transgenic mouse (the Kymouse). We show the Kymouse is susceptible to disease following infection with either H1N1, H3N2, or B/Yamagata influenza viruses and that it induces a robust binding and neutralizing antibody response to all 3 strains of influenza virus. This study demonstrates that human B-cell repertoire mice can be used for influenza virus studies, providing a tool for further interrogation of the antibody response.

## Teaser

Understanding how antibody responses evolve to influenza virus infection in humans is complex because of the variable exposure history. In the current study we investigate anti-influenza antibody responses in a mouse engineered to express the human antibody repertoire (the Kymouse).

Influenza remains a significant cause of disease and death. While there was a significant reduction in cases during the 2020–2021 and 2021–2022 seasons due to nonpharmaceutical interventions associated with the coronavirus disease 2019 pandemic, the 2022–2023 burden of influenza infection in the United States was equivalent or greater than prepandemic levels, with 27–54 million influenza-like illnesses, 290 000–650 000 hospitalizations, and 19 000–58 000 deaths [1]. The main method of reducing influenza disease burden remains vaccination. However, current influenza vaccines are imperfect [2]. Vaccine efficacy is variable each year and often quite low, from 19% in the 2014–2015 season up to 60% in the 2010–2011 season. This reflects the high variability of influenza virus and mismatches between vaccine strains and circulating virus. On top of challenges presented by seasonal influenza virus variations, completely novel influenza virus strains can emerge, causing pandemics. All of these issues with the seasonal influenza vaccine have prompted research into new approaches for vaccines with broader coverage.

While novel cassette-based vaccine platforms such as RNA may overcome some of these problems, increasing the breadth of protection offered by influenza vaccines is a research priority [3]. Immunity against influenza virus is directed against several different antigens including neuraminidase (NA), matrix 2 (M2) ectodomain, and nucleoprotein, but the major target for antibodies is hemagglutinin (HA), which is a membrane glycoprotein with 2 major domains—a head that mediates viral entry via binding sialic acids [4] and a stem. Most neutralizing antibodies bind the head region of HA; however, the head region has a high mutation rate and antibodies against the HA head of one strain do not neutralize other strains as effectively [5, 6]. Vaccines that provide protection against multiple influenza virus strains are commonly described as pan-influenza or universal. However, one potential roadblock in the development of a universal influenza vaccine is original antigenic sin (OAS) or antigenic imprinting. This was first coined by Thomas Francis in the 1960s [7], who proposed that following repeat exposure to an antigenically variable immunogen, the body will continue to recall an immune response against the “first” version of the immunogen encountered, rather than developing a new immune response against the current version. Recent advances show that influenza infection and vaccination mostly boost previous antibody responses, called back-boosting, and elicit some new neutralizing antibodies [8].

To better develop universal influenza vaccines, especially those targeting HA, more information is needed to understand the influence of OAS on immunity to HA in the context of infection and vaccination, particularly antibody evolution. Human studies are complex because of the unknown natural history of influenza exposure in participants being studied; an alternative is to use animal models. However, of the 9 animal models studies looking at this issue, 6 had evidence of OAS occurring, but 3 did not observe it [9]. A particular problem with inferring human OAS from animal models is the difference in the B-cell repertoire to humans. These differences will alter how naive B cells and antibodies bind the HA antigen and how humoral responses change over time. An approach that can overcome this problem is to use transgenic mice with humanized immunoglobulin loci [10]. The Intelliselect transgenic mouse (Kymouse) has a complete set of human variable (V_H_), diversity (D_H_), and junction (J_H_) genes at the immunoglobulin heavy locus. It also has human V and J genes inserted at both the immunoglobulin lambda (Igλ) and immunoglobulin kappa (Igκ) loci. However, the immunoglobulin constant genes (C_H_, C_λ_, and C_κ_) remain murine. The endogenous mouse loci are inactivated and replaced by these human genes. The Kymouse has a diverse B-cell repertoire with key similarities to the human repertoire [11]. It has been used to generate antibodies against human immunodeficiency virus type 1 [12], malaria [13], and severe acute respiratory syndrome coronavirus 2 [14]. In this study, we investigated responses to influenza virus infection in the Kymouse. We tested different influenza virus subtypes for their ability to infect the Kymouse and measured the resulting subtype-specific antibody response following prime-boost regimens using different influenza virus strains, demonstrating that the Kymouse is a good platform for understanding vaccine and infection responses in the context of influenza virus and the human B-cell immune repertoire.

## MATERIALS AND METHODS

### Mouse Immunization and Infection

Adult male and female Kymab mice were obtained from Kymab (Babraham, United Kingdom [UK]) and kept in specific-pathogen-free conditions in accordance with the UK's Home Office guidelines. Kymouse was originally generated by the injection of 129S7 embryonic stem cells into the C57BL/6 *Tyr^c-Brd^* mouse strain [10]; thus, it is a mixture of 129S7 and C57BL/6J genetic backgrounds.

All work was approved by the Animal Welfare and Ethical Review Board at Imperial College London under PPL P4EE85DED. Mice were maintained in autoclaved individually ventilated cages under positive pressure, with a mixture of Tapvei Eco-Pure Premium Aspen chips (Datesand) and Sizzle-Pet (1034015; LBS, UK) for bedding. Mice were housed in groups of up to 5 animals per cage. Mice had ad libitum access to irradiated RM3 pellets for food.

For infections, mice were anesthetized using isoflurane followed by intranasal application of A/California/7/2009 (H1N1), B/Florida/04/06 (B/Yamagata), or A/HKx31 (that has the HA and NA genes from A/Aichi/2/68 [H3N2] and the remaining 6 genes from A/Puerto Rico/8/34) influenza virus in a 100-µL volume. Virus was grown in Madin-Darby canine kidney cells, in serum-free Dulbecco’s modified Eagle medium (DMEM) supplemented with 1 µg/mL trypsin, and virus titer was determined by plaque assay. Mice were immunized with 50 µL containing 2.5 µg antigen and AddaVax adjuvant (1:1 v/v) in the vastus lateralis muscle.

At specified time points postimmunization, blood samples were taken by tail vein bleed and sera isolated after clotting by centrifugation. Mice were culled using 100 μL intraperitoneal pentobarbitone (20-mg dose, Pentoject, Animalcare Ltd, UK).

### Semiquantitative Antigen-Specific Enzyme-Linked Immunosorbent Assay

Antibodies specific to influenza virus were measured in sera using a standardized enzyme-linked immunosorbent assay (ELISA) [15]. For coating, MaxiSorp 96-well plates (Nunc) had 1 µg/mL antigen (all His tagged from Sino Biological): either A/California/07/2009 (11085-V08B), B/Florida/4/2006 (11053-VO8H), A/X-31 (40059-V08B), or NA from A/California/07/2009 N1 (11058-V07B); standard wells were coated with a combination of anti-murine lambda and kappa light chain–specific antibodies (AbD Serotec) and incubated overnight at 4°C. Plates were blocked with 1% bovine serum albumin in phosphate-buffered saline (PBS). Bound immunoglobulin G (IgG) was detected using horseradish peroxidase–conjugated goat antimouse IgG (AbD Serotec) and tetramethylbenzidine substrate followed by sulfuric acid as stop solution. Optical density was read at 450 nm. A dilution series of recombinant murine immunoglobulin was used as a standard to quantify specific antibodies.

### Hemagglutination Inhibition Assay

Samples were analyzed by hemagglutination inhibition (HAI) assay using A/California/07/2009 (H1N1) virus strain as described previously [16]. Serum samples were pretreated with receptor-destroying enzyme (RDE; Denka Seiken) for 18 hours at 37°C before inactivating the enzyme at 56°C for 1 hour. RDE-treated serum was 2-fold serially diluted across the plate with PBS and incubated with prediluted 4 hemagglutinating units of virus per well for 15 minutes at room temperature. One hundred microliters of 0.5% turkey erythrocytes diluted in PBS was added to each well, and plates were incubated for 30 minutes at room temperature before scoring the response.

### Pseudovirus Neutralization Assay

Hemagglutinin genes from H1N1 (A/California/07/2009) and H3N2 (A/X-31) influenza A virus and (B/Florida/4/2006) influenza B virus were cloned and ligated into pcDNA3.1(+) expression vector. HEK293T (5 × 10^4^) cells were co-transfected with 12 µg of either H1, H3, or B along with 12 µg p8.91-lentiviral vector, 18 µg pCSFLW, and 10 µg TMPRSS-2. Supernatant containing released H1, H3, or B pseudotyped particles were harvested after 24 hours, followed by centrifugation at 5000*g* for 10 minutes to remove any debris, and concentrated via ultra-centrifugation. Filtered viral supernatant were analyzed via luciferase reporter activity assay. HEK293T (10^4^) cells were seeded in a 96-well tissue culture plates (Thermo Fisher Scientific, UK) and grown until about 70%–80% confluency achieved. Heat-inactivated serum samples from immunized mice were diluted in DMEM + Tosyl phenylalanyl chloromethyl ketone (5 µg/mL). Diluted serum samples were added to the cells in the presence of H1, H3, or B pseudotyped particles at a concentration of 10^6^ relative light units in 50 µL. Plates were incubated for 48 hours at 37°C in 5% carbon dioxide. Following 48 hours’ incubation, the luciferase reported activity was read using Bright Glo (Promega, Brentford) luciferase assay system on a Microplate Luminometer.

### Statistical Analysis

Calculations as described in figure legends were performed using GraphPad Prism 9 (GraphPad Software Inc, La Jolla, California). In some instances, statistical analysis was not possible due to small group sizes.

## RESULTS

### The Kymouse Is Highly Susceptible to Influenza Infection Challenge With H1N1, H3N2, and B Strains

The study aim was to investigate responses to influenza virus antigens in the Intelliselect transgenic mouse (Kymouse). We first tested whether it was possible to infect the Kymouse with influenza virus. We compared signs of disease following infection with 3 different strains of influenza virus: clinically isolated strains of H1N1 (A/California/07/2009) and B/Yamagata (B/Florida/04/06) and a laboratory strain of H3N2 (A/HKx31, referred to as X31). We performed a dose titration starting at the doses of each virus that we have seen to give moderate disease in CB6F1 mice [17].

**Figure 1.**
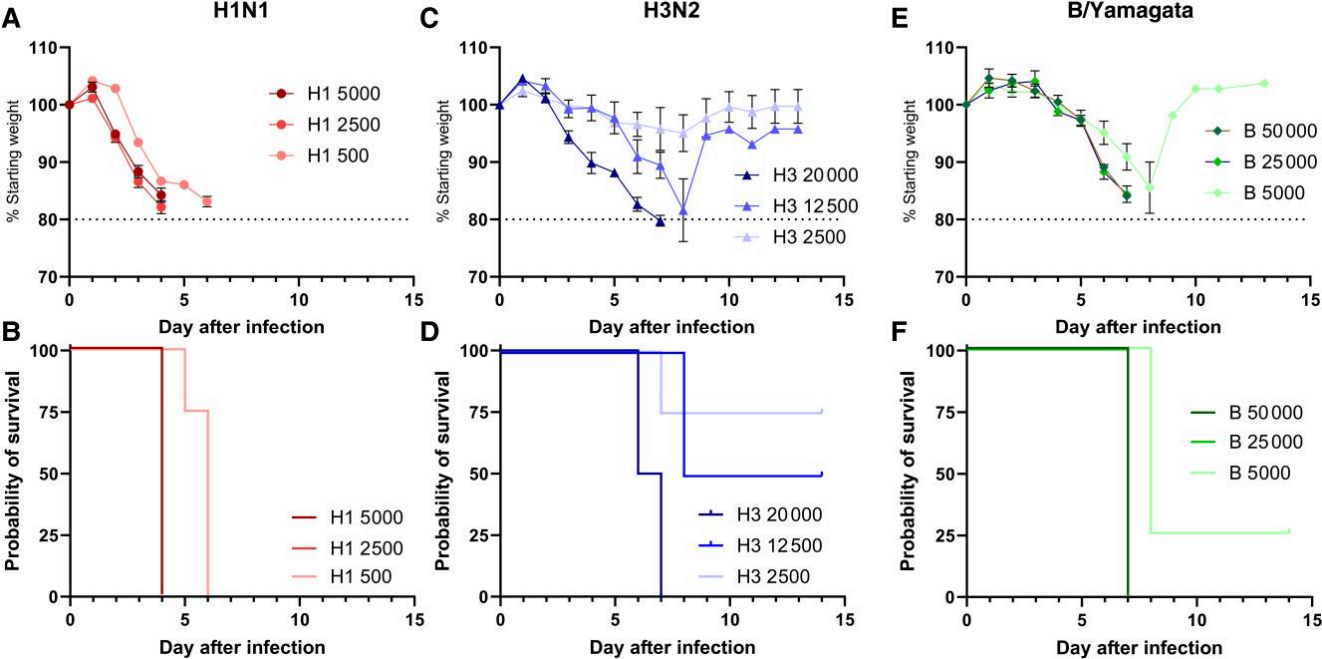
The Kymouse is highly susceptible to influenza infection challenge with H1N1, H3N2 and B strains. Kymouse were intranasally inoculated with various doses of influenza virus in 100 µl. Weight change after infection (*A*, *C*, *E*), survival after infection (*B*, *D*, *F*) with A/England/195/2009 (H1N1; *A-B*), X/31 (H3N2; *C-D*) or B/Florida/4/2006 (B/Yamagata: *E-F*) influenza. N = 4 mice per group. Points represent mean ± Standard Error of the Mean (SEM) in panels *A*, *C*, *E*.

Mice infected with H1N1 virus rapidly lost weight at all 3 doses (5000, 2500, or 500 plaque-forming units [PFU]; Figure 1*A*), necessitating humane experiment termination (endpoint) by day 6 after infection (Figure 1*B*). Mice in the H3N2 groups lost less weight (Figure 1*C*), with all mice in the 20 000 PFU group and half of the mice in the 12 500 PFU group reaching the humane endpoint (Figure 1*D*). Infection with B/Yamagata also led to rapid weight loss in the higher-PFU groups (Figure 1*E*), necessitating humane culling of all the mice receiving higher doses and 3 of 4 of the low-dose group (Figure 1*F*). Infectious dose de-escalation showed that even when H1N1 dose was reduced to 50 PFU, there was weight loss, necessitating culling at day 7 after infection (Supplementary Figure 1*A* and 1*B*). However, a 1000 PFU dose of H3N2 or a 500 PFU dose of B/Yamagata gave similar weight loss kinetics, and recovery of all animals from infection except 1 B/Yamagata animal. The data suggest that the Kymouse is susceptible to influenza virus infection using both clinical isolates and mouse-adapted strains. Based on these findings, we used 5 PFU H1N1, 1000 PFU H3N2, and 500 PFU B/Yamagata for subsequent studies.

### Prior Exposure to Influenza Provides Protection Against Subsequent Challenge With Heterologous Surface Antigens

**Figure 2.**
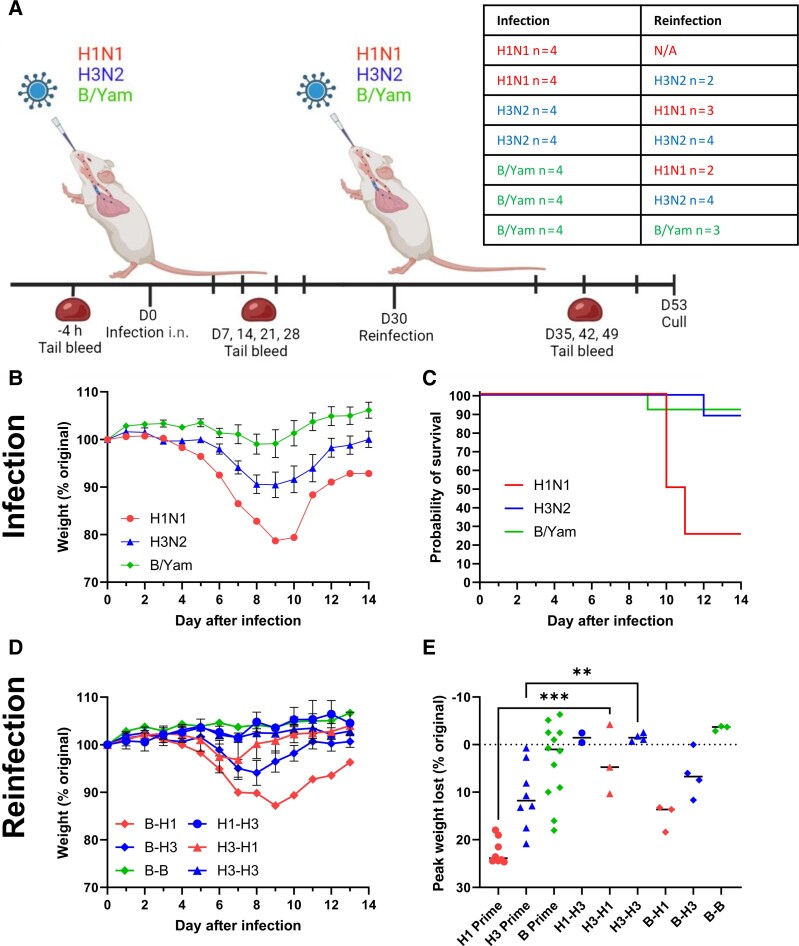
Prior exposure to influenza provides some protection against subsequent re-infection with a heterologous strain. Kymouse were intranasally inoculated with influenza virus in an infection-reinfection regime; schematic and table indicating schedule, virus and animal numbers (*A*). Weight change after infection (*B*, *D*), survival after infection (*C*, *E*). Points represent mean ± SEM (*B*, *D*). Initially n = 4 per group, some exceeded humane endpoint, numbers for analysis presented in table. ** *P* < .01, *** *P* < .001, comparison by ANOVA and post test (*E*). Panel A generated in Biorender. Abbreviations: B/Yam, influenza B/Yamagata; i.n., intranasal; N/A, Not Applicable.

We investigated if initial infection protected from subsequent infection with an influenza virus strain expressing the same or different surface antigens (Figure 2*A*). Initially, mice received a priming dose of either 5 PFU of H1N1, 1000 PFU of H3N2, or 500 PFU of B/Yamagata. Modest disease was seen in the H3N2 and B/Yamagata groups (Figure 2*B*). We still observed significant mortality after H1N1 infection and had to cull 6 of 8 animals; 1 animal was culled from each of the H3N2 and B/Yamagata groups (Figure 2*C*). Once the mice had fully recovered (day 32 after initial infection), they were infected again either with the same or a different subtype. The profile of weight loss was different to the primary infection (Figure 2*D*). Previous infection was protective against weight loss on reinfection with the homologous virus, seen for both H3N2 (H3-H3, Figure 2*D*) and B/Yamagata (B-B, Figure 2*D*). We also evaluated responses to infection with a virus with heterologous surface antigens; mice previously infected with H3N2 lost significantly less weight when challenged with H1 and vice versa, compared to previously naive mice (comparing peak weight loss for H3-H1 or H1-H3 to H1 primary infection; Figure 2*E*). It is worth noting that the H3N2 challenge virus used is chimeric, with H3N2 surface antigens, but H1N1 internal antigens, which may have affected protection. There was no protective effect of an initial B/Yamagata infection on H1 or H3 challenge. These studies indicate that homologous infection is protective against symptomatic infection (weight loss) in the Kymouse, but there was no cross-protection between influenza A and B.

### Kymouse Infected With Influenza Raise an Antibody Response

**Figure 3.**
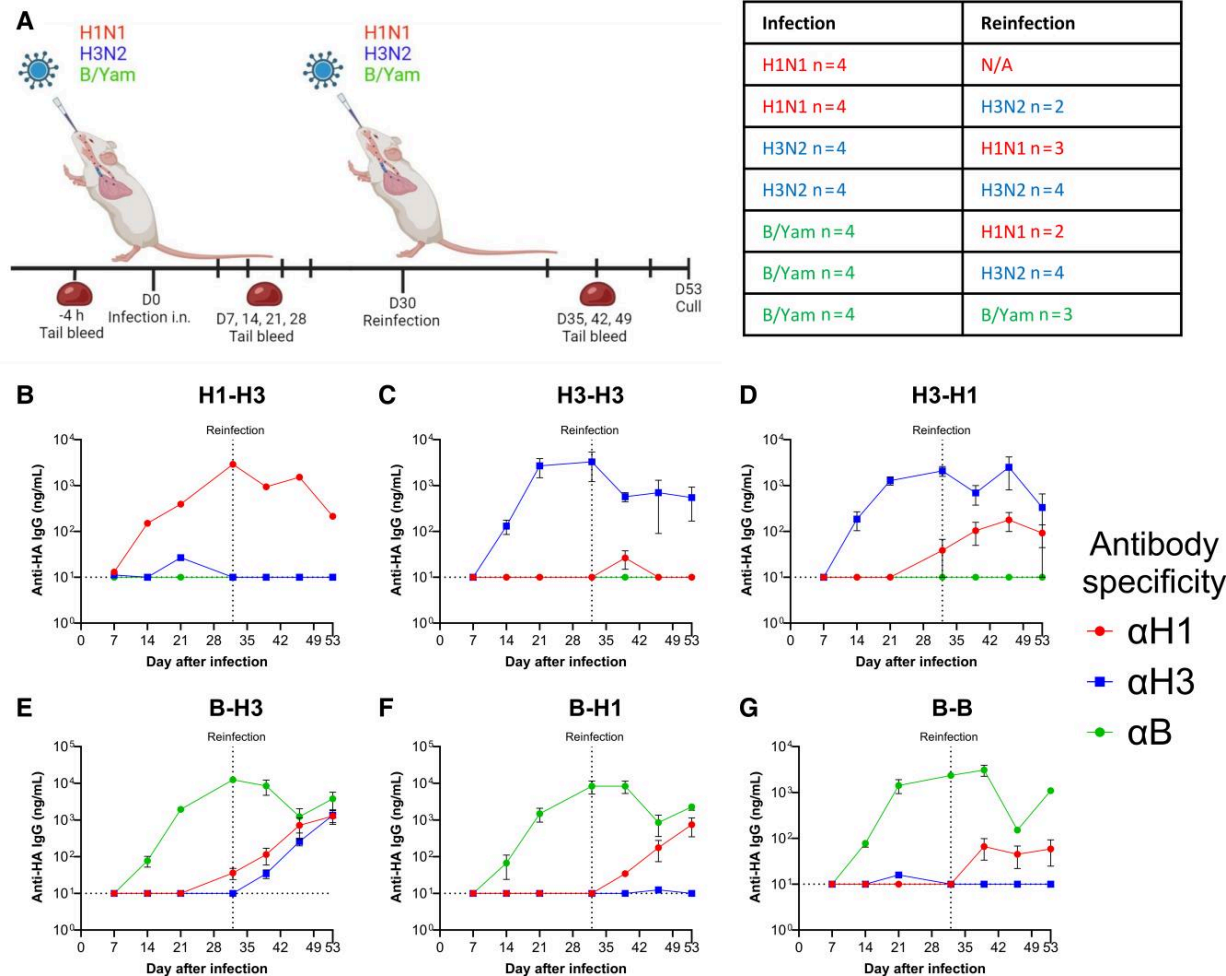
Kymouse mount a virus specific response to primary and secondary infections. Kymouse were intranasally inoculated with influenza virus in an infection-reinfection regime: same mice as Figure 2; schematic and table indicating schedule, virus and animal numbers (*A*). Anti-HA responses to H1, H3, B in each of the regimes. H1-H3 (*B*), H3-H3 (*C*), H3-H1 (*D*), B-H3 (*E*), B-H1 (*F*), B-B (*G*). Initially n = 4 per group, some exceeded humane endpoint, numbers for analysis presented in table. Panel A generated in Biorender. Points represent mean ± SEM. Abbreviations: B/Yam, influenza B/Yamagata; HA, hemagglutinin; IgG, immunoglobulin G; i.n., intranasal; N/A, Not Applicable.

We then investigated the kinetics of antibody responses against HA antigens in the Kymouse. Serum was collected weekly after the viral infections (Figure 3*A*). Antibody responses were measured using ELISA, testing for mouse-specific IgG (Kymouse Fab region is human origin, but the Fc region is murine). One limitation of the interpretation of these data is that the number of H1N1 prime animals that survived the initial infection was only 2.

Initially we compared responses against the 3 HA antigens in mice within each infection regimen. Mice infected with H1, then H3, had a robust H1 response but did not produce anti-H3 or anti-B antibodies above background (Figure 3*B*). Mice infected with a homologous H3-H3 regimen had a strong H3 response, not boosted by reinfection (Figure 3*C*). Mice infected with a heterologous H3-H1 regimen had H3 antibodies after prime and then H1 antibodies after boost (Figure 3*D*). Interestingly, mice infected with B first had detectable binding antibodies to both H3 and H1 after the H3 reinfection (Figure 3*E*). Mice infected with a B-H1 regimen also had robust B antibodies after prime, and then increased H1, but no anti-H3 on boost (Figure 3*F*). Surprisingly, mice in the B-B homologous group had an increase in anti-H1 antibodies on the B boost (Figure 3*G*).

**Figure 4.**
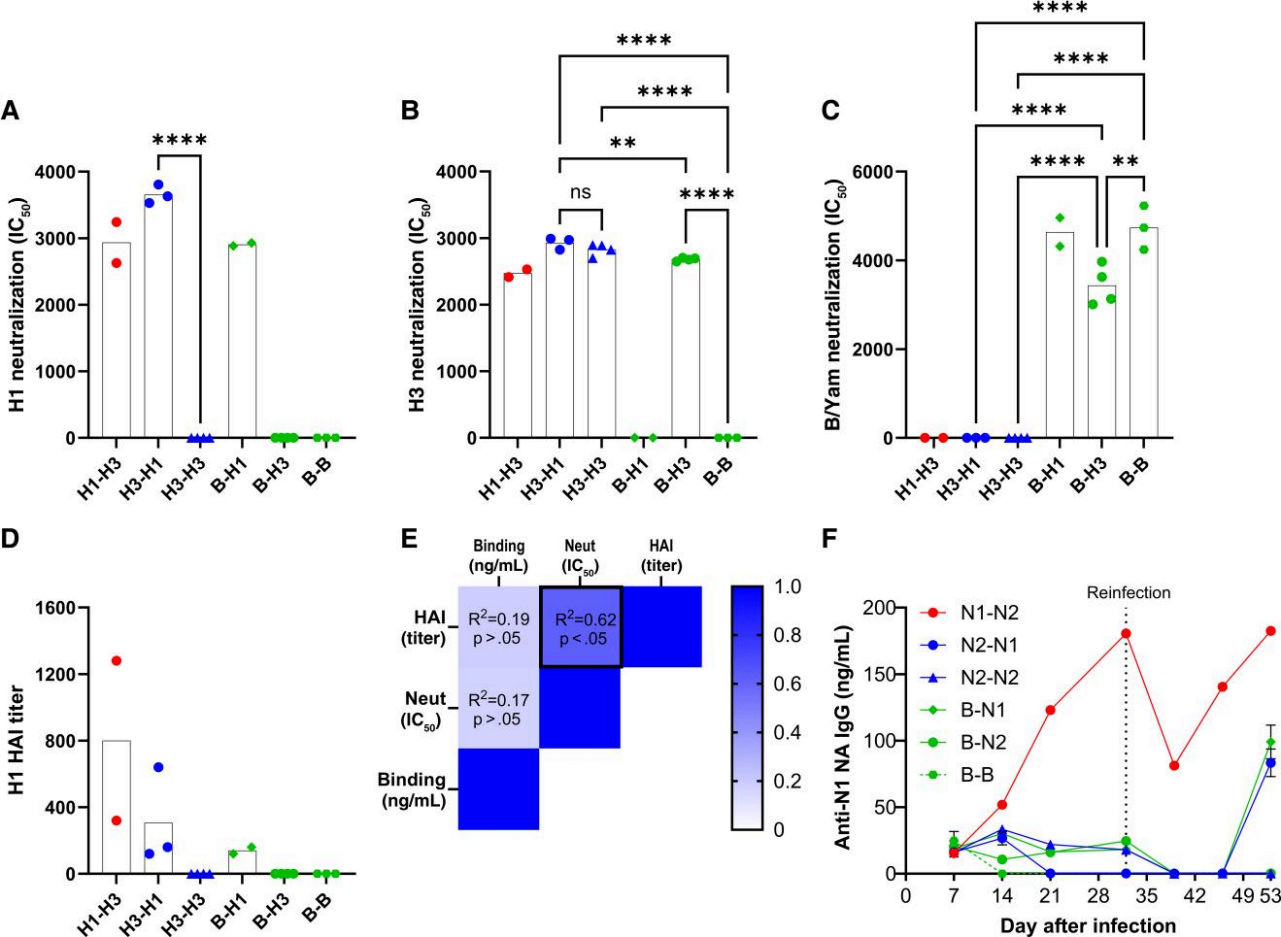
Kymouse generate influenza virus neutralising antibody following infection. Kymouse were intranasally inoculated with influenza virus in an infection-reinfection regime. Blood was collected weekly and neutralisation titre was measured by pseudotype assay for H1 (*A*), H3 (*B*) and B (*C*). HAI titre for H1 (*D*). Correlation between peak anti-H1 HA binding ELISA, H1 Neutralisation IC50 and HAI (*E*). Anti-N1 NA antibody titre (*F*). Points represent individual animals (*A–D*, *F*) and mean ± SEM (*F*); animal numbers as per Figures 2 and 3. Abbreviations: B/Yam, influenza B/Yamagata; HAI, hemagglutination inhibition; IC50, half-maximal inhibitory concentration; IgG, immunoglobulin G; NA, neuraminidase; ns, not significant.

Virus neutralization titers were assessed at day 53 after the start of the study by pseudovirus neutralization assay. Mice that had any exposure to H1N1 virus had high H1 neutralizing titers (Figure 4*A*); this was significantly higher than mice not exposed to H1. H3 pseudovirus neutralization titers were detectable in mice that had been infected with H3N2 (Figure 4*B*); these were significantly higher than those in mice not exposed to H3N2. B pseudovirus neutralization titers were significantly higher in the animals infected with B/Yamagata (Figure 4*C*). There was a detectable H1 HAI titer in the H1-exposed animals (Figure 4*D*). We compared the 3 H1 antibody assays; there was no significant correlation between binding ELISA and HAI or neutralization titer, but a significant correlation between HAI and neutralization (*R*^2^ = 0.62, *P* > .05; Figure 4*E*). We also measured responses to the other major surface antigen of influenza, NA. We only measured NA-specific antibodies from the N1 strain (we did not look at N2 or B NA). After the initial infection, only mice infected with H1N1 had a detectable N1 response (Figure 4*F*). On boosting, the groups that received H1N1 on second infection also developed an anti-N1 NA response. These data suggest that the order of exposure to influenza strains can impact the antibody response to subsequent viruses in the Kymouse.

### Influenza Immunization Raises an Antibody Response

**Figure 5.**
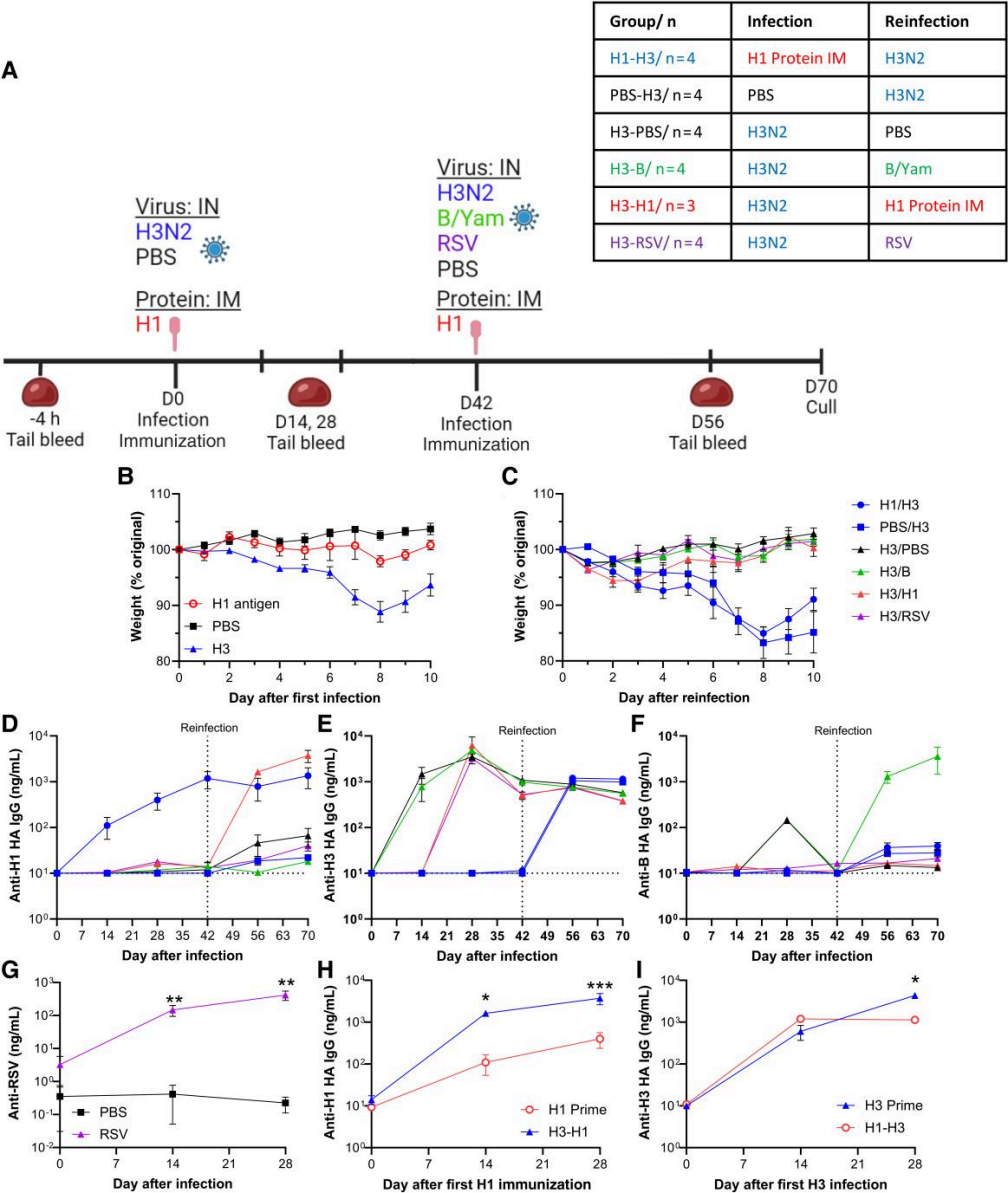
Kymouse mount an antibody response to vaccination and infection. Kymouse were intranasally inoculated with influenza virus, PBS or RSV in an infection-reinfection regime; two groups also received 2.5 µg H1 HA with AddaVax adjuvant intramuscularly; schematic of study and group sizes (*A*). Weight change after infection (*B*, *C*). Blood was collected weekly and anti-HA IgG responses measured for H1 (*D*), H3 (*E*), B (*F*) or RSV (*G*). Comparison of responses from time of first exposure to either H1 (*G*) or H3 (*H*). Points represent mean ± SEM. Initially n = 4 per group, some exceeded humane endpoint, numbers for analysis presented in table. Abbreviations: B/Yam, influenza B/Yamagata; HA, hemagglutinin; IgG, immunoglobulin G; IM, intramuscular; IN, intranasal; PBS, phosphate-buffered saline; RSV, respiratory syncytial virus.

We determined the effect of immunization or infection (Figure 5*A*) on subsequent heterologous infection and heterologous reinfection. Mice were immunized with H1 HA antigen intramuscularly with the AddaVax adjuvant. There was no weight loss after immunization or intranasal PBS delivery (Figure 5*B*), but H3 infection led to weight loss, peaking on day 9. Forty-two days after the first infection/immunization, mice had a secondary influenza antigen exposure by infection or immunization. To determine whether a completely unrelated infection had any impact on the antibody response, one group of mice was challenged with a low dose of respiratory syncytial virus (RSV). Mice that had not previously been exposed to H3 lost weight on secondary infection with H3N2 (Figure 5*C*). At the dose used, RSV did not cause weight loss in the Kymouse, and influenza virus B secondary infection caused no weight loss.

We collected serum every 14 days during the study and evaluated antibody binding responses to HA from the different virus strains. Responses to H1 were seen following immunization, either as a primary exposure or as boost in the group after H3 infection prime (Figure 5*D*). There were some low-level responses to H1 HA in the H3-infected animals, but not in the PBS group. H3 infection before H1 immunization did not alter the overall anti-H1 titer on boost. When looking at anti-H3 responses, there was some variability between mice infected with H3N2 at day 14, but by day 28 all mice had a strong response to the first H3N2 infection. Subsequent infection with B, RSV, or H1 had no impact on anti-H3 titer (Figure 5*E*). Mice given PBS or H1 immunization first had no detectable anti-H3 HA antibody after prime. However, on H3 infection, both groups developed an anti-H3 response. The only group that responded to influenza B was the group that had H3 then B infection (Figure 5*F*). The H3-RSV group had detectable anti-RSV IgG at 14 and 28 days after infection (Figure 5*G*). Looking at the kinetics of the response relative to first exposure to antigen, H3 prime infection increased the response to H1 immunization (Figure 5*H*); there is an indication the H1 immunization reduced the peak response to H3 (Figure 5*I*).

## DISCUSSION

In this study, we investigated influenza virus infection and resulting antibody responses in the human-like antibody repertoire of the Kymouse. The overall aim of the study was to determine whether the Kymouse would respond to influenza virus infection or immunization by generating strain-specific antibody responses. We observed high susceptibility to influenza virus infection in these mice, with severe disease necessitating culling at very low infectious doses of virus, particularly H1N1. Once we had titered the virus to a dose that could be survived, we assessed antibody responses to the different strains H1N1, H3N2, and B/Yamagata. Infection protected against severe disease from homologous reinfection and strain-specific binding/neutralization could be detected.

One of the striking features was the severity of influenza in this mouse strain. We saw significant weight loss necessitating humane culling with very low doses of virus even compared to our previous studies [17]. This was particularly the case with H1N1. The H1N1 virus used was not mouse adapted but derived from a clinical strain isolated during the 2009 pandemic (A/California/07/2009). The disease is most probably determined by the underlying mouse strain: The Kymouse is a mixed 129S7 and C57BL/6J background. Mouse strain has considerable impact on susceptibility to influenza infection, and C57BL/6 is one of the more susceptible strains [18]. Despite this underlying infection susceptibility, primary infection was protective against homologous challenge. We were not able to further investigate the cause of weight loss and mortality because of the small number of animals and the difference in disease kinetics (with endpoints being at different times after initial infection). Our study and other previous studies suggest a role for viral-induced cytokines in disease following infection in mice [19]; this is something that needs further investigation. Whether the underlying strain has an impact or the genetic modification also needs further investigation, comparing wild type to transgenic animals.

There were some differences seen in the effect of primary exposure on the outcome of a second infection using a virus with heterologous surface antigens. Though numbers were small, H1N1 infection protected against H3N2 challenge and mice lost significantly less weight on H1N1 infection after a primary H3N2 infection; whether this is antibody mediated or T-cell based is unclear, as the X31 strain has internal genes from an H1 strain (PR8), which can provide cross-protection [20]. Prior immunization with H1 antigen, while it did produce an antibody titer, did not protect against H3 infection, suggesting that antibody alone was not cross-protective. One intriguing result was a reduction in the reinfection response after a closely related initial infection, so H1 first reduced H3 boost. However, the cause of this is unclear—it may be that the protection provided against the shared internal antigens between H1N1 and X31 prevents productive infection and therefore reduces seroconversion. A recent study used molecular tools to tag B cells to track the order of exposure [21]; they observed that when very closely related sequences were used, the existing response can suppress the development of de novo antibody response, but that this was related to the proximity of the prime and boost antigens.

Our study is an observational study with constraints on interpretation stemming from the limited numbers of animals used. A limited amount of statistical analysis has been performed where group sizes permitted, but the high mortality caused by the challenge viruses (especially the H1N1) affected our ability to evaluate differences between regimens. It should be noted that while the H1N1 and B strains are clinical isolates, the H3N2 virus is an older laboratory strain; we have previously observed that clinical isolates of H3N2 are poorly infectious in mice [17]. For future studies, immunizing with antigens from current circulating H3 strains may be a way to get more reflective responses, as we show that the mice respond well to H1 immunization.

Protection from homologous and heterologous virus infection following immunization or a primary infection suggests the Kymouse can be used to dissect responses to related antigens and to observe how human repertoires develop. Having titered the virus to a dose at which the mice can recover from challenge means the mice can be used for future challenge studies to test vaccines, especially when considering the impact of antigen design and OAS. Infection and immunization generated virus-specific antibodies and, given the human B-cell repertoire, the Kymouse provides a tractable model to study immune imprinting and immunization on a complex preimmune background.

## Supplementary Data

Supplementary materials are available at *The Journal of Infectious Diseases* online (http://jid.oxfordjournals.org/). Supplementary materials consist of data provided by the author that are published to benefit the reader. The posted materials are not copyedited. The contents of all supplementary data are the sole responsibility of the authors. Questions or messages regarding errors should be addressed to the author.

## Supplementary Material

jiae472_Supplementary_Data

## Data Availability

Data available on request from the corresponding author.

## Notes

***Acknowledgments.*** The authors thank Stefania Scalabrino, Siobhan O’Leary, and Sarah Smith for project management support; Carolyn Newell and Yvette Ighorue for financial and administration support; Kasia S and Andrew G for assistance in the in vivo studies; and Lesley Rawlinson for laboratory management. For the purpose of open access, the author has applied a Creative Commons Attribution (CC BY) license to any Author Accepted Manuscript version arising. The Kymouse is available through collaboration with Sanofi or LifeArc, but acquisition needs to be initiated separately of the corresponding author.

***Author contributions*.** V. M.: Data curation, investigation. S. J. W.: Resources, conceptualization. R. F. C.: Investigation. N. J. T.: Resources. S. T. R.: Conceptualization, acquisition, writing–review and editing. P. K.: Conceptualization, funding acquisition, writing–review and editing. J. S. T.: Conceptualization, funding acquisition, investigation, writing–original draft.

***Financial support*.** This study was supported by a grant from the Bill & Melinda Gates Foundation to Kymab (grant number OPP1159947).
