# Supplementary material for: A Transgenic Mouse With a Humanized B-Cell Repertoire Mounts an Antibody Response to Influenza Infection and Vaccination
Source: J Infect Dis. 2024 Sep 24;231(2):e299–307. doi: 10.1093/infdis/jiae472 (PMC11841647; doi:10.1093/infdis/jiae472)
Supplement: jiae472_Supplementary_Data [file jiae472_supplementary_data.pdf]

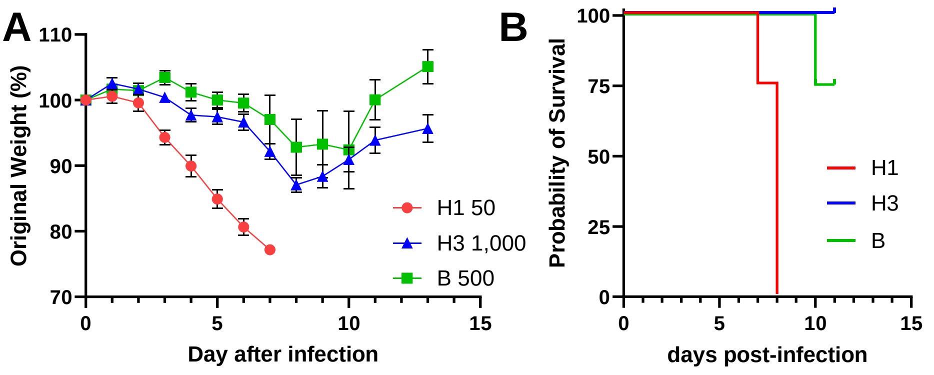

Figure S1: **Influenza virus causes disease at extremely low doses.** Kymouse were infected with A/England/195/2009 (5 PFU), X/31 (1,000 PFU) or B/Florida/4/2006 (500 PFU) influenza viral strains. Weight change after infection (A), survival after infection (B). N=4 mice per group. Points represent mean +/- SEM.
